# Can ChatGPT explain it? Use of artificial intelligence in multiple sclerosis communication

**DOI:** 10.1186/s42466-023-00270-8

**Published:** 2023-08-31

**Authors:** Hernan Inojosa, Stephen Gilbert, Jakob Nikolas Kather, Undine Proschmann, Katja Akgün, Tjalf Ziemssen

**Affiliations:** 1 Center of Clinical Neuroscience, Department of Neurology, University Hospital Carl Gustav Carus, Technische Univesität Dresden, Fetscherstr. 74, 01307 Dresden, Germany; 2 Else Kröner-Fresenius Center for Digital Health, Faculty of Medicine Carl Gustav Carus, Technische Universität Dresden, Dresden, Germany

Dear editor

Use of artificial intelligence (AI) is rapidly becoming an important resource in medical care, promising potential advantages and support for a more efficient practice in the coming years. Recently, large language models (LLMs) have shown the ability of generating high-quality texts simulating human language [1]. These models have attracted considerable attention and stimulated a discussion of their potential use among the general population, researchers and medical professionals, highlighting the possible opportunities of these technologies [2]. As we experience a new era of AI applications, it is important to explore the use of such models in various aspects of healthcare, from diagnostics to patient education, potentially qualifying them as medical device, but also to discuss their potential risks and regulatory challenges [3–5]. Regulatory oversight is in the interlinked areas of data security, the assessment and approval requirements when LLM are used for a medical intended purpose and the AI-safeguarding requirements being introduced in the EU and other jurisdictions for foundation models applied in high-risk settings.

A debate and evaluation of the performance of ChatGPT, a LLM chatbot by OpenAI (San Francisco, California, USA), is occurring practically at every level of the society [6]. The latest version of the OpenAI generative pre-trained transformer 4 (GPT-4), was recently launched with impressing abilities of synthesizing natural language, compared to previous LLMs. Similar as in several other medical fields and neurological diseases, this type of technology could offer a support in the management of patients with multiple sclerosis (MS).

MS affects particularly young people (and more frequently women) that are at the peak of their productive age. It is therefore natural that this population may seek for an explanation or a “second opinion” regarding several aspects of their disease as they frequently have an active participation in decision-taking. Neurological concepts underlying MS and therapeutic approaches are complex and patients ought to look for an explanation in online resources beyond their medical consultations, even though mainstream information sources may be difficult to understand [7]. Likewise, using openly accessible LLM chatbots, such as ChatGPT, could be an attractive source of information for patients about their disease. This could also enhance the practice of healthcare professionals caring for MS patients, as it may support the bidirectional communication between patients and practitioners, particularly in primary care.

Explorative reports have demonstrated an impressive, albeit not flawless, ability from ChatGPT in explaining findings in medical reports for patients [8]. Other potential opportunities and uses in medicine are currently under discussion such as writing of medical reports, note-taking, writing of scientific papers or even conducting consultations or therapy in specific fields [9–11]. However, as far as we are aware, no specific in-depth analysis or clinical validation regarding the quality of generated texts in relation to management of MS is so far available. This is particularly important, as the text corpus on which ChatGPT is trained is not specific for medical or neurological texts. Although Open AI has not disclosed the specific data sources, these come from diverse settings in the internet, including websites (e.g. posts from social media), books or other documents. Other LLMs have been developed based on biomedical literature or clinical notes, although using a smaller database such as e.g. BioBERT [12], BLURB [13] or BioMegatron [14] but these are not as popular and accessible as ChatGPT.

Moreover, several limitations and flaws have been also described for natural language models, including ChatGPT. Of special importance is the tendency to “hallucinate” text based on inexistent information, with the risk of misleading medical doctors or patients if this information is misinterpreted [15, 16]. Additionally, if the information provided by the LLM is used for interpretation of data (e.g. medical exams, findings) and support of medical decision-taking, there is potential for harm, as inaccuracies or misinterpretations could impact the safety and outcomes of patients.

Other important aspect is the readability of texts generated by LLMs. General information regarding MS in frequent online resources tends to require a relatively high educational level for complete understanding [7]. This does not correspond with recommendations from the requirements from the National Institute of Health (NIH) in the USA. Here, a 6th -7th grade-reading level is recommended (approx. age 11–12 years) for patient-facing information. Using LLM with specific instructions for these groups may be better suited to these patients.

We conducted an exploratory study of the use of ChatGPT for communication of important medical information and concepts in a range of MS scenarios. We tested the humanness, correctness and relevance of texts from ChatGPT using the GPT4-model. Additionally, we assessed a parallel set of generated texts in a more understandable patient-focused language as a supportive communication tool for patients.

We manually generated and tested a series of 64 frequent medical concepts and scenarios relevant in the treatment of MS patients and asked ChatGPT to define or explain them based on limited inputs. A prompt-based instruction was initially provided, and two texts were generated for each scenario: one addressed to general practitioners and one for MS patients (Fig. 1). The second report had to be readable by populations with a sixth grade of education or over. Beyond text generation, ChatGPT nor other LLM were used for writing of this manuscript.

**Fig. 1:**
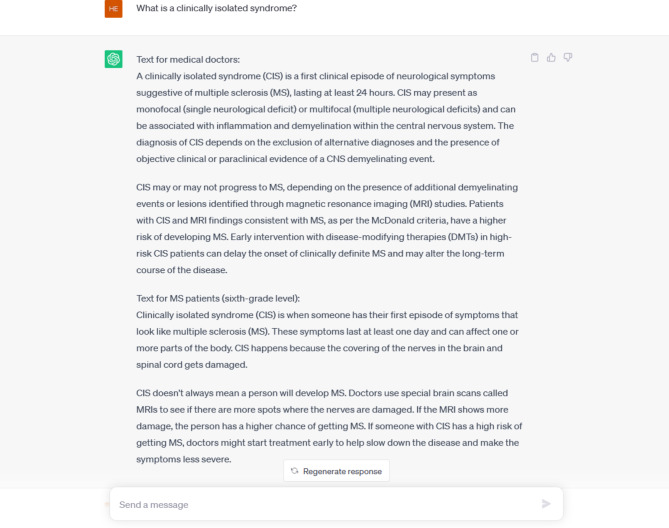
Example of generated texts for medical doctors and patients and corresponding input Note: example of texts generated by ChatGPT in one particular scenario. An initial instruction for generation of two texts (one focused on medical doctors, one for patients readable with a sixth grade level) was provided.

Texts included the following categories: (i) explaining the MS diagnosis and relapses; (ii) treatment options or indication; and, (iii) disease monitoring and considerations regarding family planning that are frequently topic of MS consultations. We also asked for an explanation of the mechanism of action, administration and safety control of several disease modifying treatments (DMTs). Finally, we addressed specific questions regarding current MS research or specific treatment situations. These were evaluated independently by three medical doctors with experience in care of MS patients at the MS Centre of the University Hospital Dresden (HI, TZ, KA). Humanness, correctness, and relevance were reviewed and scored for each report generated by ChatGPT on a Likert scale ranging from 1 to 5 (1 = not human-like, correct or relevant at all; 2 = slightly human-like, correct or relevant; 3 = somewhat human-like, correct or relevant, 4 = highly human-like, correct or relevant and 5 = completely human-like, correct or relevant). Humanness referred to terms of style, tone and expression; the medical accuracy and reliability of the texts were considered in the correctness; for relevance, we considered the alignment of the texts with the subject matter, considering focus and purpose of provided information. Writing-quality of ChatGPT-generated reports was also assessed with established standardized scoring systems. We included the Flesch-Kincaid Reading Ease index measures readability based on syllable count and sentence length, with higher scores being easier to read. The Fleisch-Kincaid Grade Level, the Gunning Fog Index, Simple Measure of Gobbledygook (SMOG) and the Colemann Liau score were used to estimate the approximate educational grade level required to understand the text [17–21]. Data were analyzed with descriptive statistics and non-parametric tests to assess differences according to target groups of the texts and category of the scenarios using IBM SPSS statistics 28.0.1.0 (142).

The overall analysis of the 128 generated medical texts (64 for medical doctors and 64 for patients) showed very high humanness with a median score of 5 (range 4–5) and mean of 4.95 (SD 0.15) points (Table 1). Correctness had a median value of 4.25 (range 2–5) and mean of 4.15 (SD 0.58). Relevance rating had a median score of 4 (range 3–5) and mean of 4.20 (SD 0.47). Chi-square tests revealed no differences in humanness, correctness or relevance of texts addressed to medical professional and patients. These were also similar if the texts addressed MS definitions and diagnosis, DMT or other aspects (*data not shown*). Although no comparison was made with texts written directly by healthcare professionals, texts generated by ChatGPT were considered by the assessors to be human-like, almost in their totality. The main goal of this chatbot, generating human-like conversations, was also achieved in the MS jargon.

**Table 1:** Characteristics of multiple sclerosis specific texts generated by ChatGPT for medical doctors and patients

	Total texts	Texts for medical doctors	Texts for patients	*p*-value
N	128	64	64	
Humanness, median (range)	5 (4–5)	5 (4–5)	5 (4–5)	0.130
Correctness, median (range)	4.25 (2–5)	4.5 (2–5)	4 (2–5)	0.976
Relevance, median (range)	4 (3–5)	4 (3–5)	4 (3–5)	0.808
Flesch-Kincaid readability index, mean (SD)	39.19 (25.83)	15.26 (8.86)	63.14 (10.11)	< 0.001
Fleisch-Kincaid grade level, mean (SD)	12.83 (4.02)	16.45 (2.01)	9.23 (1.50)	0.003
Gunning fog, mean (SD)	15.99 (4.62)	20.17 (2.18)	11.83 (1.75)	0.001
Coleman-Liau index, mean (SD)	14.68 (4.06)	18.31 (2.05)	11.04 (1.47)	0.001
Simple Measure of Gobbledygook index, mean (SD)	11.50 (3.48)	14.57 (1.76)	8.44 (1.50)	< 0.001

Note: this table reflects the obtained evaluations of humanness, correctness and relevance of multiple sclerosis texts generated by ChatGPT as evaluated by three medical doctors and readability scores

While most texts were highly or completely correct, certain mistakes occurred leading to scores between 2 and 3 points. When discussing possible options for mild relapsing remitting MS (RRMS), a therapy with fingolimod was mentioned together with other DMTs such as interferon or glatiramer acetate as first-option drugs, although it is more frequently recommended in cases with highly active course [22]. Regional differences in therapy selection should be considered, as this may differ among countries and regulatory agencies [22]. Similarly, siponimod was considered as a first option for RRMS while it is rather used for active secondary progressive MS. Probably, training dataset played an important role here, as the Food and Drug Administration (FDA) and European Medicines Ageny (EMA) differ slightly in the label. Other therapeutic errors were observed, such as: (i) a titration with an initial dose of 7 mg teriflunomide was recommended; and, (ii) dimethyl fumarate was provided as an on-label option for patients with clinically isolated syndrome (CIS).

We also observed that ChatGPT made recommendations that may not be standard-of-care in the monitoring of MS, such as using gadolinium in imaging at a regular basis and as a routine to detect inflammation [23]. Another aspect that was not completely correct was the recommendation of optional laboratory controls in patients treated with fingolimod or teriflunomid, as these should be routinely performed to monitor adverse reactions.

ChatGPT could effectively adjust to a language suitable to medical doctors or patients as demanded. The mean Flesch-Kincaid readability index was 15.26 (SD 8.86) and 63.14 (SD 10.11), respectively. The word selection and construction of sentences were therefore in the texts addressed to patients easier to read. A required education corresponding approx. to an 8th grade level was obtained in the Fleisch-Kincaid grade level for MS patients, while a 16th grade was obtained for medical doctors. Similarly, an estimation of more educational years was obtained for professional texts compared to those with a simpler language using the Gunning fog, SMOG or Coleman-Liau index. Comparisons between both groups reflected significant Chi-Square tests for these scores.

In this brief exploratory analysis, ChatGPT seems to excel at an adaptive use of MS terminology for communication between neurologists and other healthcare professionals, as well as with MS patients. As the text corpus of the current models is not finely adjusted to specific MS scenarios, reliability of outputs by ChatGPT was, however, not completely accurate. Specific errors were observed, especially in important aspects related to the immune therapy and disease monitoring. A potential patient harm of using this or other chatbots without medical supervision is evident, especially being easily and openly accessible on the web and an attractive option for a “second opinion”. As a small group of MS-specialized doctors performed the qualitative analysis, further examinations including a larger and more heterogeneous group of raters, including e.g. doctors without MS specialization or general practitioners and patients with different education levels seem necessary. An evaluation of human-generated texts could also bring further inside regarding the performance of this (an other) LLMs.

Additionally, using a specific dataset with scientific literature (e.g. medical guidelines, scientific biomedical literature) or considering specific (e.g. regional) differences in care could attenuate the observed errors. An interesting approach could be a further fine-tuning of these models with specific datasets (e.g. from MS centers), so these adapt to the standards of care in clinical practice. This is possible, although limited for ChatGPT and very flexible with several open-source models (e.g. LLaMA and the fine-tuned versions Alpaca or Vicuna [24, 25]).

Currently, although texts generated through LLM are almost completely human-like, they are not entirely accurate. Certain information could lead to patient harm if not corrected by expert care providers. For this reason, thorough validation of LLMs in appropriate contexts for their use cases is required to ensure patient safety. Contrary to thoughts part of certain discussions, LLM could complement, albeit not replace, professional expertise in this context. However, with improved accuracy and validation, LLMs could further provide a quick and cost-effective solution for communication with other physicians and patients. Proper supervision from qualified personal and regulatory agencies is to date necessary.

## Data Availability

All data are available from the corresponding author.

## References

[1] Singhal, K. (2022). *Large Language Models Encode Clinical Knowledge* arXiv preprint arXiv:2212.13138.

[2] Lee P, Bubeck S, Petro J (2023). Benefits, limits, and risks of GPT-4 as an AI Chatbot for Medicine. New England Journal of Medicine.

[3] Ordisch, J. (2023). *Large Language Models and software as a medical device*

[4] Union, T. E. P.a.t.c.o.T.E. (2017). *Regulation (EU) 2017/745 of the European Parliament of the COuncil of 5 April 2017 on medical devices*. Official Journal of the European Union.

[5] Haupt CE, Marks M (2023). AI-Generated medical Advice—GPT and Beyond. Journal Of The American Medical Association.

[6] Bubeck, S. (2023). *Sparks of artificial general intelligence: Early experiments with gpt-4* arXiv preprint arXiv:2303.12712.

[7] Moccia M (2016). Can people with multiple sclerosis actually understand what they read in the internet age?. Journal of Clinical Neuroscience.

[8] Jeblick, K. *ChatGPT Makes Medicine Easy to Swallow: An Exploratory Case Study on Simplified Radiology Reports* arXiv preprint arXiv:2212.14882, 2022. 10.1007/s00330-023-10213-1 PMC11126432 37794249

[9] Patel SB, Lam K (2023). ChatGPT: The future of discharge summaries?. The Lancet Digital Health.

[10] Digital TL (2023). ChatGPT: Friend or foe?. The Lancet Digital Health.

[11] Else H (2023). Abstracts written by ChatGPT fool scientists. Nature.

[12] Lee J (2019). BioBERT: A pre-trained biomedical language representation model for biomedical text mining. Bioinformatics.

[13] Gu, Y. (2021). *Domain-specific Language Model Pretraining for Biomedical Natural Language Processing*. *ACM Trans Comput Healthcare*, 3(1): p. Article 2.

[14] Shin, H. C. (2020). *BioMegatron: Larger biomedical domain language model* arXiv preprint arXiv:2010.06060.

[15] Alkaissi H, McFarlane SI (2023). Artificial Hallucinations in ChatGPT: Implications in Scientific writing. Cureus.

[16] Ji, Z. (2023). *Survey of Hallucination in Natural Language Generation*. *ACM Comput Surv*, 55(12): p. Article 248.

[17] Coleman M, Liau TL (1975). A computer readability formula designed for machine scoring. Journal of Applied Psychology.

[18] Flesch R (1948). A new readability yardstick. Journal of applied psychology.

[19] Mclaughlin, G. H. (1969). *SMOG grading - a New Readability Formula*. The Journal of Reading.

[20] Gunning R (1969). The fog index after twenty years. Journal of Business Communication.

[21] McLaughlin, G. (1969). *SMOG grading–A new readability formula in the journal of reading*. May.

[22] Ayzenberg I, Hoepner R, Kleiter I (2016). Fingolimod for multiple sclerosis and emerging indications: Appropriate patient selection, safety precautions, and special considerations. Therapeutics And Clinical Risk Management.

[23] Wattjes MP (2021). 2021 MAGNIMS-CMSC-NAIMS consensus recommendations on the use of MRI in patients with multiple sclerosis. Lancet Neurology.

[24] Chiang, W. L. L. (2023). Zhuohan and Lin, Zi and Sheng, Ying and Wu, Zhanghao and Zhang, Hao and Zheng, Lianmin and Zhuang, Siyuan and Zhuang, Yonghao and Gonzalez, Joseph E. and Stoica, Ion and Xing, Eric P., *Vicuna: An Open-Source Chatbot Impressing GPT-4 with 90\%* ChatGPT Quality*

[25] Hashimoto, R.T.a.I.G.a.T.Z.a.Y.D.a.X.L.a.C.G.a.P.L.a.T.B., (2023). *Stanford Alpaca: An instruction-following LLaMA model*. GitHub repository.

